# A Meta-Analysis of Effects of Bt Crops on Honey Bees (Hymenoptera: Apidae)

**DOI:** 10.1371/journal.pone.0001415

**Published:** 2008-01-09

**Authors:** Jian J. Duan, Michelle Marvier, Joseph Huesing, Galen Dively, Zachary Y. Huang

**Affiliations:** 1 Ecological Technology Center, Monsanto Company, St. Louis, Missouri, United States of America; 2 Environmental Studies Institute, Santa Clara University, Santa Clara, California, United States of America; 3 Department of Entomology, University of Maryland, College Park, Maryland, United States of America; 4 Department of Entomology, Michigan State University, East Lansing, Michigan, United States of America; University of Zurich, Switzerland

## Abstract

**Background:**

Honey bees (*Apis mellifera* L.) are the most important pollinators of many agricultural crops worldwide and are a key test species used in the tiered safety assessment of genetically engineered insect-resistant crops. There is concern that widespread planting of these transgenic crops could harm honey bee populations.

**Methodology/Principal Findings:**

We conducted a meta-analysis of 25 studies that independently assessed potential effects of Bt Cry proteins on honey bee survival (or mortality). Our results show that Bt Cry proteins used in genetically modified crops commercialized for control of lepidopteran and coleopteran pests do not negatively affect the survival of either honey bee larvae or adults in laboratory settings.

**Conclusions/Significance:**

Although the additional stresses that honey bees face in the field could, in principle, modify their susceptibility to Cry proteins or lead to indirect effects, our findings support safety assessments that have not detected any direct negative effects of Bt crops for this vital insect pollinator.

## Introduction

Currently, all commercialized genetically engineered insect resistant crops are based on crystalline (Cry) proteins encoded by genes derived from the soil dwelling bacterium *Bacillus thuringiensis* (Bt). Studies on the mode of action and toxicology of Bt Cry proteins have established that these proteins are toxic to select groups of insects [1]–[4]. Cry proteins currently produced in commercialized Bt crops target insects in the orders Lepidoptera (moths) and Coleoptera (beetles). Because of this specificity, most experts feel it is unlikely that these Bt crops would impact honey bee (Hymenoptera: *Apis mellifera* L.) populations [e.g., 5], [6]. Nevertheless, because of their importance to agriculture – the economic value of honey bee pollination for U.S. agriculture has been estimated to be worth $0.15–19 billion per year [7] – honey bees have been a key test species used in environmental safety assessments of Bt crops [8], [9]. These assessments have involved comparisons of honey bee larval and adult survival on purified Cry proteins or pollen collected from Bt crops versus survival on non-Bt control material.

To date, no individual tests involving Bt crops or Cry proteins that target Lepidoptera or Coleoptera have shown significant impacts on honeybees [1], [6]. Despite this, there have been suggestions in the popular press that Bt proteins produced in insect resistant crops might be contributing to recent declines in honeybee abundance [10], [11]. Given this speculation about potential adverse impacts of Bt crops on honeybees and the possibility that small sample sizes may have undermined the power of prior risk assessment experiments (Table 1: studies to date have rarely employed more than 2–6 replicates per treatment), a formal meta-analysis, combining results from existing experiments, may provide more definitive answers. Meta-analysis increases statistical power and can reveal effects even when each of the individual studies failed to do so due to low replication [12], [13]. A recent meta-analysis, synthesizing results from 42 field studies involving Bt cotton and maize [14], did not examine effects on honey bees because very few studies have reported field data for this group [but see 15]. Here we report a meta-analysis of 25 laboratory studies (Table 1) that focused on the chronic and/or acute toxicity of Bt Cry proteins or Bt plant tissues (pollen) on honey bee larvae and adults.

**Table 1:** Major characteristics of the laboratory studies included in the meta-analysis.

Ref #	peer reviewed	Cry protein	target	Cry protein source	[Cry]	exposed stage	exposure method	control	response variable	*n*	control mean	exp. mean	control SD	exp. SD
[15] *	yes	Cry1Ab	Lepidoptera	corn pollen	not specified	adults	GM corn pollen	non-GM corn pollen	survival	6	44.00	44.00	30.437	36.420
[15] *	yes	Cry1Ab	Lepidoptera	corn pollen	not specified	adults	5 g cakes 80% GM corn pollen: 20% honey (w/w)	5 g cakes 80% non-GM corn pollen: 20% honey (w/w)	survival	10	71.40	82.00	17.551	9.202
[21]	yes	Cry3B	Coleoptera	GM *E. coli*	0.066% soln.	larvae	Cry protein in sugar soln.	sugar soln.	survival	2	100.00	98.82	0.000	1.680
[21]	yes	Cry3B	Coleoptera	GM *E. coli*	0.332% soln.	larvae	Cry protein in sugar soln.	sugar soln.	survival	2	100.00	98.82	0.000	1.680
[22]	yes	Cry1Ab	Lepidoptera	corn pollen	not specified	larvae	1.5 mg GM maize pollen in 50% sugar soln.	1.5 mg non-GM maize pollen in 50% sugar soln.	larval mortality	5	3.07	3.07	6.858	1.715
[22]	yes	Cry1Ab	Lepidoptera	corn pollen	not specified	larvae	1.5 mg GM maize pollen in 50% sugar soln.	1.5 mg non-GM maize pollen in 50% sugar soln.	larval mortality	5	5.50	3.74	1.969	3.446
[22]	yes	Cry1Ab	Lepidoptera	corn pollen	not specified	larvae	1.5 mg GM maize pollen in 50% sugar soln.	1.5 mg non-GM maize pollen in 50% sugar soln.	pupal mortality	3	2.87	2.87	3.861	1.103
[22]	yes	Cry1Ab	Lepidoptera	corn pollen	not specified	larvae	1.5 mg GM maize pollen in 50% sugar soln.	1.5 mg non-GM maize pollen in 50% sugar soln.	pupal mortality	3	5.11	3.07	0.885	3.099
[22]	yes	Cry1F	Lepidoptera	corn pollen	not specified	larvae	1.5 mg GM maize pollen in 50% sugar soln.	1.5 mg non-GM maize pollen in 50% sugar soln.	larval mortality	5	3.07	4.60	6.858	3.858
[22]	yes	Cry1F	Lepidoptera	corn pollen	not specified	larvae	1.5 mg GM maize pollen in 50% sugar soln.	1.5 mg non-GM maize pollen in 50% sugar soln.	larval mortality	5	5.50	6.16	1.969	4.430
[22]	yes	Cry1F	Lepidoptera	corn pollen	not specified	larvae	1.5 mg GM maize pollen in 50% sugar soln.	1.5 mg non-GM maize pollen in 50% sugar soln.	pupal mortality	3	2.87	4.46	3.861	2.758
[22]	yes	Cry1F	Lepidoptera	corn pollen	not specified	larvae	1.5 mg GM maize pollen in 50% sugar soln.	1.5 mg non-GM maize pollen in 50% sugar soln.	pupal mortality	3	5.11	5.62	0.885	3.542
[23]	yes	Cry1Ac	Lepidoptera	cotton pollen	not specified	adults	0.16 g GM cotton pollen in 0.4 ml 50% sucrose soln.	0.16 g non-GM cotton pollen in 0.4 ml 50% sucrose soln.	mortality	4	1.25	3.75	2.250	2.250
[24]	no	Cry1Ac	Lepidoptera	B.t.k.	20 ppm	adults	Cry protein in honey: water soln.	honey: water soln.	mortality	3	24.76	23.93	4.640	10.430
[25]	no	Cry1Ac	Lepidoptera	B.t.k.	20 ppm	larvae	Cry protein in honey: water soln.	honey: water soln.	survival (dosed to emerge.)	4	81.50	87.50	19.824	16.114
[25]	no	Cry1Ac	Lepidoptera	B.t.k.	20 ppm	larvae	Cry protein in honey: water soln.	honey: water soln.	survival (emerge. to term.)	4	66.38	57.73	26.206	38.980
[26]	no	Cry3A	Coleoptera	B.t.t.	100 ppm	adults	Cry protein in honey: water soln.	honey: water soln.	mortality	3	29.92	25.01	7.400	2.860
[27]	no	Cry3A	Coleoptera	B.t.t.	100 ppm	larvae	Cry protein in distilled water	distilled water	survival	4	82.50	86.00	8.062	19.114
[28]	no	Cry3A	Coleoptera	B.t.t.	100 ppm	larvae	Cry protein in DI water	DI water	mortality	4	12.50	20.00	13.229	21.602
[29]	no	Cry3Bb1	Coleoptera	B.t.	1.79 mg/ml	larvae	Cry protein in DI water	DI water	mortality	4	2.50	0.00	2.890	0.000
[30]	no	Cry3Bb1	Coleoptera	B.t.	0.36 mg/ml	adults	Cry protein in 30% sucrose: DI water soln.	30% sucrose: DI water soln.	mortality	4	43.80	40.37	15.186	3.687
[31]	no	Cry1F	Lepidoptera	corn pollen	5.6 mg/ml	larvae	transgenic pollen in sucrose soln.	non-transgenic pollen in sucrose soln.	survival	4	98.75	97.50	2.500	5.000
[31]	no	Cry1F	Lepidoptera	GM *P. fluore-scens*	640 ng/bee	larvae	Cry protein in sucrose soln.	non-GM pollen in sucrose soln.	survival	4	98.75	92.50	2.500	11.902
[32]	no	Cry2Ab2	Lepidoptera	B.t.	68 µg/ml	adults	Cry protein in sodium carbonate soln. w/ 30% sucrose	sodium carbonate soln. w/ 30% sucrose	mortality	4	21.67	19.04	5.755	2.785
[33]	no	Cry2Ab2	Lepidoptera	B.t.	170 µg/ml	larvae	Cry protein in sodium carbonate buffer	sodium carbonate buffer in DI water	mortality	4	7.50	11.25	6.455	9.465
[34]	no	Cry2Ab2	Lepidoptera	B.t.	100 µg/ml	larvae	Cry protein in sodium carbonate buffer	sodium carbonate buffer in DI water	mortality	4	21.25	18.75	19.738	31.192
[35]	no	Cry3Bb1	Coleoptera	GM *E. coli*	2.59 mg/ml	adults	Cry protein in buffer in 30% sucrose soln.	buffer in 30% sucrose soln.	mortality	4	29.40	34.40	8.969	11.709
[36]	no	Cry3Bb1	Coleoptera	GM *E. coli*	2.55 mg/ml	larvae	Cry protein in buffer	buffer	survival	4	93.75	97.50	2.500	2.887
[37]	no	Cry1Ab	Lepidoptera	B.t.k.	20 ppm	adults	Cry protein in honey: water soln.	honey: water soln.	mortality	3	22.28	16.20	6.368	6.162
[38]	no	Cry1Ab	Lepidoptera	B.t.k.	20 ppm	larvae	Cry protein in honey: water soln.	honey: water soln.	survival (dosed to capped)	3	82.67	79.33	14.742	16.166
[38]	no	Cry1Ab	Lepidoptera	B.t.k.	20 ppm	larvae	Cry protein in honey: water soln.	honey: water soln.	survival (emerg. to term.)	3	96.18	91.62	5.036	6.668
[39] *	yes	Cry1Ba	Lepidoptera	B.t.	4% of total protein	adults	Cry protein in honey bee diet	honey bee diet	survival (lifespan)	3	37.03	47.17	8.385	3.968
[40] *	yes	Cry1Ba	Lepidoptera	B.t.	0.625 mg/g	adults	Cry protein in honey bee diet	honey bee diet	survival	20	91.06	89.88	12.903	13.776
[41] *	yes	Cry1Ba	Lepidoptera	B.t.	0.625 mg/g	adults	Cry protein in honey bee diet	honey bee diet	survival	9	82.43	78.72	12.980	18.360
[42]	no	Cry9C	Lepidoptera	corn pollen	5.8 µg/L	adults	GM maize pollen in honey	non-GM maize pollen in honey	mortality	6	18.00	23.33	7.483	8.914
[43] **	yes	Cry1Ac	Lepidoptera	GM *E. coli*	20 µg/ml	adults	Cry protein in 50% (v/v) honey: water soln.	50% honey: water soln.	mortality	3	25.00	24.00	43.301	42.708
[43] **	yes	Cry1Ac	Lepidoptera	GM *E. coli*	20 µg/ml	larvae	Cry protein in distilled water	distilled water	mortality (dosed to capped)	4	17.00	20.00	37.563	40.000
[44] **	yes	Cry2A	Lepidoptera	GM *E. coli*	50 µg/ml	adults	Cry protein in 30% (w/v) fructose: water soln.	30% fructose: water soln.	mortality	3	20.30	24.30	40.223	42.890
[44] **	yes	Cry2A	Lepidoptera	GM *E. coli*	50 µg/ml	larvae	Cry protein in distilled water	distilled water	mortality	4	16.30	12.50	36.937	33.072

* data obtained directly from author.

** standard deviation was calculated based on the binomial distribution as 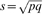, where *p* is the proportion of individuals surviving and *q* = 1−*p*.

## Methods

### Searching

To locate studies of the nontarget effects of Bt crops for honey bees, we used multiple search criteria (e.g. *Apis mellifera*/honey bees and *Bt*/*Bacillus thuringiensis*) in the online databases Agricola, BioAbstracts, PubMed, and ISI Web of Science. Additional studies were found by searching the reference lists of empirical and review papers, performing general internet searches, and sending a list of references accompanied by a request for additional suggestions to over 100 researchers who are knowledgeable about studies of nontarget effects of Bt crops. Requests were also made under the US Freedom of Information Act to obtain relevant studies submitted by industry scientists to the US Environmental Protection Agency.

### Selection

Studies had to meet a series of criteria in order to be included in this analysis. Specifically, studies had to: (i) involve Bt Cry proteins that are either lepidopteran-active (Cry1, Cry2, or Cry9 class) or coleopteran-active (Cry3 class) and that were either expressed in Bt plant tissues or produced by genetically modified *B. thuringiensis*, *Escherichia coli*, or *Pseudomonas fluorescens* strains (i.e. we excluded studies testing formulations of whole or lysed *B. thuringiensis* bacterial cells or spores, which might contain a mixture of different toxins, surfactants, and inert carrier ingredients) (ii) measure the effects of ingestion of the cry protein for honey bees of the species *Apis mellifera*; (iii) have occurred in a laboratory setting; (iv) report survival (or conversely mortality) as a response variable; (v) include a comparison to a non-transgenic control (typically sugar water or, for tissue studies, pollen from a non-transgenic plant variety); (vi) present treatment means, accompanied by standard deviations (*s*) and sample sizes (*n*) (or the author directly provided these values to us) necessary to calculate the metric of effect size, Hedges' *d* [16] (i.e., we required *n*_1_>0, *n*_2_>0, *n*_1_+*n*_2_>2, and *s*_1_(*n*_1_−1)+*s*_2_(*n*_2_−1)>0); and (vii) have been written in English. Measures of standard error, 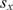, were transformed to standard deviations (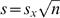) as needed. Available studies reported a range of response variables including survival, growth, development, and abundance. We focused only on survival (or mortality) data to maximize consistency among studies and reduce issues of non-independence when studies reported multiple metrics for the same sets of bees. Application of these criteria yielded data from a suite of 25 suitable publications or reports (Table 1). The Cry proteins used in these studies include those intended for use primarily in Bt corn, cotton, and potato. For those studies reporting data for multiple concentrations of a particular Cry protein, we included data for only the highest reported dosage. If data were reported as repeated measures over time for a particular life history stage (e.g. the number of adult bees alive on each of 14 days following dosage), we included data for only the final time point. Applying these criteria, in combination with the fact that several studies reported multiple independent experiments or measures of survival for multiple stages, yielded a total of 39 independent assessments of the effects of Bt proteins on honeybee survival (Table 1).

### Data abstraction

For each study, we recorded details about the Cry protein and its origin, the dose and duration of exposure, and the control treatment. When necessary, we scanned data figures and used Adobe Photoshop software to extract means and measures of within treatment variance. Authors provided raw data in several instances (noted in Table 1).

### Quantitative data synthesis

Hedges' *d* was calculated for each study as the difference between the means of the Bt Cry protein and control treatments divided by the pooled standard deviation and weighted by the reciprocal of the sampling variance [16]. The sign of Hedges' *d* was reversed for studies that reported mortality rather than survival. Negative values therefore indicate lower survival (whereas positive values indicate higher survival) in Bt Cry protein treatments compared to non-Bt control treatments. Bias-corrected bootstrapped 95% confidence intervals (CIs) were used to determine if specific effect sizes differed significantly from zero. Within group and between group heterogeneities were calculated using fixed effects models in MetaWin v.2 [17]. Fixed effects models are generally considered to be inferior due to their bias toward finding effects (Type I bias) [18]. However, Type I error is not an issue for any of our findings (see below), and we used this model deliberately to make the analysis less conservative in case *Bt* has weak effects. Moreover, mixed models collapse to fixed models when no variation remains after accounting for differences among groups and sampling error [19], and this was the case for all of the analyses presented here.

## Results

**Figure 1:**
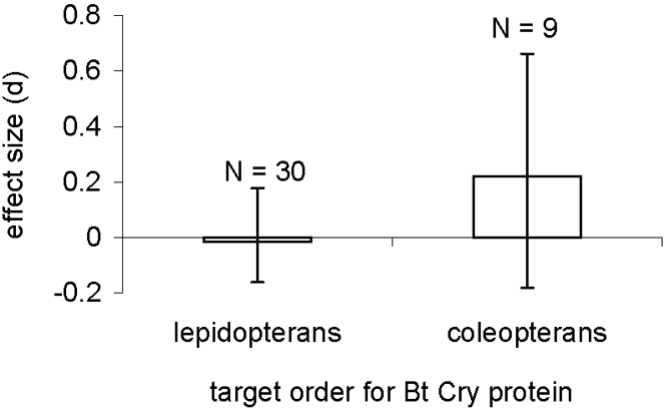
Meta-analysis of studies that report survival of honey bees exposed to Bt Cry proteins or plant tissues (pollen) that are active against lepidopterans and coleopterans. Effect size is Hedge's *d,* and error bars represent bias-corrected bootstrap 95% confidence intervals. Positive mean effect sizes indicate improved survival when exposed to Cry proteins compared to water or sugar-water control treatments. N = number of lines of independent data summarized by each bar.

When all studies were combined, no statistically significant effect of Bt Cry protein treatments on survival of honey bees was detected (N = 39, *d* = 0.025, 95% CI = −0.128 to 0.171). When data for lepidopteran-active and coleopteran-active Bt Cry proteins were compared using a fixed categorical meta-analysis model, the above pattern of no significant effects held true for each class of protein (Fig. 1). No significant difference in effect sizes was detected between lepidopteran-active and coleopteran-active proteins (*Q* = 0.668, df = 1, *P* = 0.25); nor was any significant within-group heterogeneity detected for effect sizes calculated for either lepidopteran-active (*Q*_w_ = 12.828, df = 29, *P*>0.99) or coleopteran-active proteins (*Q_w_* = 5.893, df = 8, *P* = 0.66). Mean effect sizes also did not differ (*Q* = 0.012, df = 1, *P* = 0.90) between studies that were peer-reviewed (N = 20, d = 0.015, 95% CI = −0.153 to 0.245) versus not peer-reviewed (N = 19, d = 0.039, 95% CI = −0.190 to 0.293).

**Figure 2:**
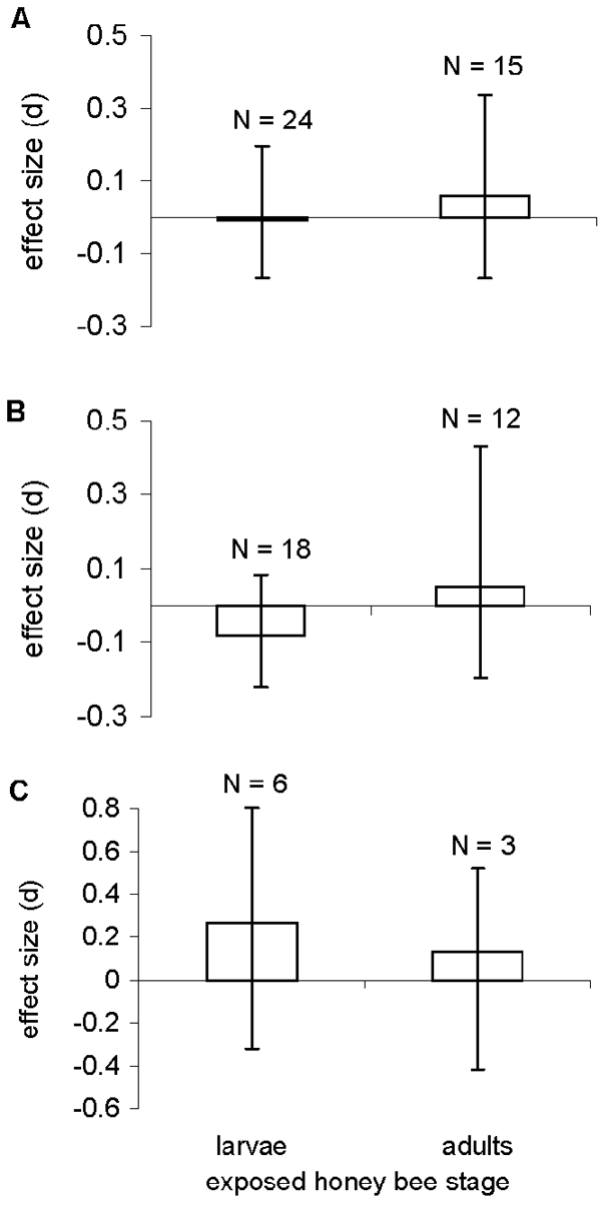
Comparison of effect sizes for larval and adult honey bees exposed to different Bt Cry proteins or plant tissues: (A) lepidopteran-active and coleopteran-active proteins combined, (B) lepidopteran-active Bt Cry proteins only, and (C) coleopteran-active protein only. Error bars and N are as described for Figure 1.

No significant effects on survival occurred with either larval or adult stages. This pattern was consistent when data from studies using lepidopteran-active and coleopteran-active Bt Cry proteins were analyzed either together (Fig. 2a) or separately (Fig. 2b&2c). No significant differences in effect sizes were detected between larvae and adults in any of the above analyses (Fig. 2a: *Q* = 0.093, df = 1, *P* = 0.69; Fig. 2b: *Q* = 0.298, df = 1, *P* = 0.47; Fig. 2c: *Q* = 0.064, df = 1, *P* = 0.80), nor were any significant within-group heterogeneities detected for the effect sizes calculated for either larvae (Fig. 2a: *Q*_w_ = 9.523, df = 23, *P*>0.99; Fig. 2b: *Q_w_* = 3.875, df = 17, *P*>0.99; Fig. 2c: *Q*_w_ = 4.746, df = 5, *P* = 0.45) or adults (Fig. 2a: *Q*_w_ = 9.772, df = 14, *P* = 0.78; Fig. 2b: *Q_w_* = 8.656, df = 11, P = 0.65; Fig. 2c: *Q_w_* = 1.084, df = 2, P = 0.58).

## Discussion

The lack of adverse effects of Bt Cry proteins on both larval and adult honey bees is consistent with prior studies on the activity-spectrum and mode of action of different classes of Bt Cry proteins. To date, with the exception of a possible ant-specific Cry22 toxin patent application, no class of Bt Cry protein has been found to be directly toxic to hymenopteran insects [4]. Although studies of acute toxicity performed in a laboratory setting may overlook sub-lethal or indirect effects that could potentially reduce the abundance of honeybees in a field setting, our findings strongly support the conclusion that the Cry proteins expressed in the current generation of Bt crops are unlikely to have adverse direct effects on the survival of honey bees. Additional analyses that included all available performance variables (survival, growth and development) similarly showed no adverse effect of Bt treatments. We do not report these results in depth here because they are potentially compromised by issues of non-independence – it is inappropriate to simultaneously include multiple measures taken on the same groups of bees. Unfortunately, few studies reported performance measures other than survival, and this prevented us from conducting separate analyses on these aspects of performance.

Although only laboratory data are synthesized here, the overall finding of no effect is consistent with the data available from a recent, well-replicated field study [15]. Additionally, the fact that laboratory studies typically expose honey bees to doses of Cry proteins that are ten or more times those encountered in the field provides additional reassurance that toxicity in the field is unlikely. However, the need for additional studies in the field may be warranted if stressors such as heat, pesticides, pathogens, and so on are suspected to alter the susceptibility of honey bees to Cry protein toxicity.

Assessment of the potential risks of Bt crops for honey bees has become increasingly refined over time. However, these studies continue to be characterized by the use of very low replication with potentially limited statistical power. Based on retrospective power analyses of their data, Rose et al. [15] recommend that “laboratory studies to measure adult bee survival should test at least six cohorts of 50 bees per treatment to detect a 50% reduction with 80% statistical power.” However, this level of replication is 1.5–3 times greater than that used in many of the similar studies performed to date (Table 1). Modest increases in the replication of these and similar studies examining potential adverse effects of transgenic crops would likely help to improve public confidence in findings of no effect [20]. In addition, meta-analysis of data from available studies testing similar hypothesis is an effective tool for quantitatively synthesizing the collective evidence regarding the safety of genetically modified crops.
